# A model for integrating strategic planning and competence-based curriculum design in establishing a public health programme: the UNC Charlotte experience

**DOI:** 10.1186/1478-4491-7-71

**Published:** 2009-08-11

**Authors:** Michael E Thompson, Andrew Harver, Marquis Eure

**Affiliations:** 1 Department of Public Health Sciences, The University of North Carolina at Charlotte, Charlotte, NC, USA; 2 Ryan White Program Part A, Mecklenburg County Health Department, Charlotte, NC, USA

## Abstract

**Introduction:**

The University of North Carolina at Charlotte, a doctoral/research-intensive university, is the largest institution of higher education in the Charlotte region. The university currently offers 18 doctoral, 62 master's and 90 baccalaureate programmes. Fall 2008 enrolment exceeded 23 300 students, including more than 4900 graduate students. The university's Department of Health Behavior and Administration was established on 1 July 2002 as part of a transformed College of Health & Human Services.

**Case description:**

In 2003, the Department initiated a series of stakeholder activities as part of its strategic planning and programmatic realignment efforts. The Department followed an empirically derived top-down/bottom-up strategic planning process that fostered community engagement and coordination of efforts across institutional levels. This process culminated in a vision to transform the unit into a Council on Education for Public Health accredited programme in public health and, eventually, an accredited school of public health. To date, the Department has revised its Master of Science in health promotion into an Master of Science in Public Health programme, renamed itself the Department of Public Health Sciences, launched a Bachelor of Science in Public Health major, laid plans for a doctoral programme, and received accreditation from the Council on Education for Public Health as a public health programme. Furthermore, the campus has endorsed the programme's growth into a school of public health as one of its priorities.

**Discussion and Evaluation:**

It is only through this rigorous and cyclical process of determining what society needs, designing a curriculum specifically to prepare graduates to meet those needs, ensuring that those graduates meet those needs, and reassessing society's needs that we can continue to advance the profession and ensure the public's health. Community stakeholders should be active contributors to programme innovation. Lessons learnt from this process include: being connected to your community and knowing its needs, being responsive to your community, remembering that process is as important as product, and preparing for success.

**Conclusion:**

The efforts reported here can be informative to other institutions by exemplifying an integrated top-down/bottom-up process of strategic planning that ensures that a department's degree programmes meet current needs and that students graduate with the competences to address those needs.

## Introduction

Events of the last decade have sparked renewed public interest in the process of promoting and protecting the public's health. Among these concerns is that less than 30% of the public health workforce has formal training in public health [1], including 75% of the heads of city or county health departments [2]. This challenge is compounded by estimates that 50% of federal and 25% of state public health workers will likely retire within the next five years [2,3].

A number of initiatives address aspects of this complex issue, including professional development opportunities for the public health workforce [4-6]; professional credentialling of public health graduates [7] that complements existing specialty certifications such as the Community Health Education Specialist (CHES) [8], the Industrial Hygienist [9] and the Registered Environmental Specialist/Registered Sanitarian [10]; evidence-based professional practice guidelines [11]; and quality assurance and accreditation mechanisms for public health departments and related agencies [12-14].

**Figure 1:**
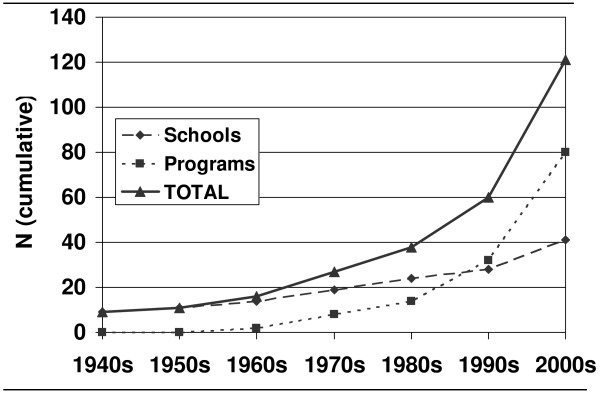
**CEPH accredited schools and programmes by decade of initial accreditation (cumulative) Source: , July 2009**.

One response to this growing demand for a trained, competent workforce has been the rapid proliferation of programmes and schools of public health accredited by the Council on Education for Public Health (CEPH) [15]. As summarized in Figure 1, the cumulative number of accredited programmes has nearly tripled over the past decade.

The number of accredited schools has also increased substantially, with some, such as those at the University of Arizona [16] and George Washington University [17], evolving from accredited public health programmes and others being created de novo as schools, such as those launched at Texas A&M University [18] and the University of Arkansas [19], with several more schools under rapid development (e.g. the University at Buffalo School of Public Health and Health Professions; East Tennessee State University College of Public Health; the University of Maryland, Baltimore, School of Public Health) [20-22].

It was within this environment of rapid change, pressing need and opportunity that the growth and development of the Department of Health Behavior and Administration (renamed the Department of Public Health Sciences in May 2007) within the College of Health and Human Services at the University of North Carolina at Charlotte (UNC Charlotte) coalesced. UNC Charlotte, a doctoral/research-intensive university, celebrated its 60th anniversary in September 2006. It is the fourth-largest of the 17-constituent University of North Carolina system and the largest institution of higher education in the Charlotte region.

The university comprises seven professional colleges and currently offers 18 doctoral programmes, 62 master's degree programmes and 90 programmes leading to bachelor's degrees. Fall 2008 enrolment exceeded 23 300 students, including more than 4900 graduate students.

The expansion of UNC Charlotte's graduate programmes in the past 10 years as well as the fortuitous location of the university (in a city where the health care industry is one of the area's largest employers [23] and within a county that is home to the state's largest health department [24]) contributed to a major restructuring of the former College of Nursing and Health Promotion into the College of Health and Human Services in 2002. The new College was proposed in a summary report submitted to the Provost on 26 November 2001, after several years of related activities, including: review during the 2002–2007 academic planning cycle of the Report of the Health Commission (submitted on 10 July 2000); a campus-wide conversation surrounding "behavioral health" (held in Spring 2001); and, a consensus recommendation by the Task Force on Behavioral Health (delivered on 1 October 2001) [25].

The final proposal included the formation of a School of Nursing, transfer of the Department of Social Work from the College of Arts & Sciences, and a restructuring of the former Department of Health Promotion and Kinesiology into two separate units, the Department of Health Behavior & Administration and the Department of Kinesiology.

On 22 March 2002, the UNC Charlotte Board of Trustees approved implementing a range of strategic initiatives related to the establishment of the College of Health and Human Services. In time, new priorities emerged from these combined efforts, including the development of graduate and undergraduate public health degree programmes, CEPH accreditation and plans for a school of public health.

## Case description

**Figure 2:**
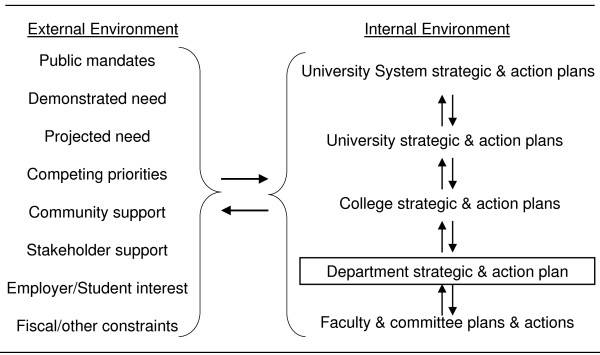
**Hierarchy of mutually reinforcing strategic and action plans**.

Like many institutions, the University of North Carolina system engages in an interactive and iterative systematic strategic and action planning process [26]. Through critical assessment of data, input from higher and lower administrative units and inputs from stakeholders and others in the external environment, each unit develops a mutually reinforcing strategic and action plan [27-29]. At the department and programme level, the iteration of this strategic planning process involved the classic SWOT (strengths, weaknesses, opportunities and threats) analysis leading to changes in mission and priorities (Figure 2).

**Figure 3:**
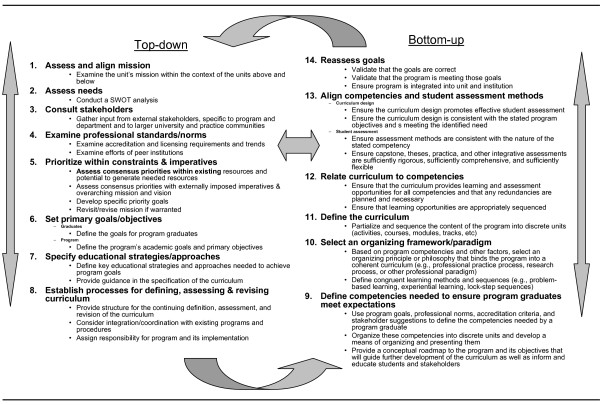
**Top-down/bottom-up strategic planning process**.

These decisions, in turn, precipitate a top-down/bottom-up planning and action cycle (Figure 3). The top-down portion reflects the mandates and expectations imposed on the department from higher units within the university system as well as external stakeholders such as accrediting bodies, employers and students. When reaching the faculty level, these tasks become distributed among a number of evidence-gathering and consensus-building efforts, both internal and external.

The empirically derived top-down/bottom-up cycle will be described concurrently with its application to this case. While presented linearly, the process, in reality, is iterative and cross-linked throughout. Information and ideas generated at one step may require revisiting decisions made in earlier steps before resuming the process. Likewise, many of these process steps are pursued simultaneously (by different or the same individuals) and information is shared, allowing for ongoing self-informing and self-correcting activities.

### Top-down

While not always well stated or made explicit, the process employed in the Department has, more or less, followed this concurrent top-down/bottom-up strategic planning approach.

### Assess and align mission

The newly created Department of Health Behavior and Administration was charged with administering two existing graduate degree programmes (Master of Science in Health Promotion and Master of Health Administration), several Certificate programmes, and an Interdisciplinary Health Studies undergraduate minor. During its first year, the Department's research, teaching and service agendas converged into a focused and integrated vision under the theme "public health and health behaviour outcomes from a social-ecological perspective."

By design, the Department had been organized as an interdisciplinary unit intended to overcome the often artificial, discipline-specific barriers to research and academic programming [30]. Consequently, the new Department was uniquely positioned to examine a wide range of possible goals, initiatives and programmes in light of its new environment.

The process started by simultaneously examining faculty strengths, community priorities and explicit university and college goals [31] to frame discussions that began at the unit's first retreat in Spring 2002. The university had recently dedicated itself to transforming from a comprehensive university to a research-oriented university. The newly conceived College of Health and Human Services was perceived as a potential leader in establishing a thriving research base that emphasized population health and health behaviour research while generating responsive and progressive health and human service training programmes. The development of the Department's mission was quickly synchronized with the institution's rapid growth, the rich resources of the Charlotte-Mecklenburg region, and national trends evident, for example, in recent Institute of Medicine reports [32,33].

### Assess needs

The faculty initiated a detailed needs assessment. This assessment process drew upon prior needs assessments and related information collected during the formation of the new College of Health and Human Services. The SWOT analysis catalogued many of the region's existing health resources and the significant contribution of health and human services to the local economy. The analysis documented that Charlotte was one of the largest United States cities not served by either a school of medicine or a school of public health [34].

### Consult stakeholders

A seminal community roundtable was held in 2003, with representation from a broad spectrum of stakeholders from professional practice (both governmental and nongovernmental), academic and community settings. Guided by a professional facilitator, three key themes emerged from this roundtable. First, there was broad support for reorienting the existing MS in Health Promotion from a focus on health education and worksite wellness into a broader public health degree.

The Master of Science in Public Health (MSPH) emerged as a favoured idea over the Master of Public Health (MPH) because of the expressed need for competent practitioners versed in research methods and translation of research findings into programmatic activity. The practitioners from the community, operating in an environment facing the pressures of evidence-based practice, translational research, imminent accreditation of public health agencies and the likely credentialling of public health practitioners, strongly supported the "S" in MSPH, believing that the combination of letters conveyed their desired emphasis on both the practice of public health and the science of public health research. Stakeholders further suggested that offering the MSPH might serve to distinguish the programme from public health programmes available in the region.

Second, there was broad support to expand the focus of the department beyond health behaviour and administration into all public health disciplines and to establish undergraduate and doctoral programmes in public health that would serve the needs of state and local agencies and allow the programme to grow into a full-fledged school of public health. The third recommendation was that the Department institutionalize and nurture its new-found connection to the practice community through the establishment of an advisory board.

### Examine professional standards/norms

In considering the academic and resource implications of these stakeholder recommendations, the Department sought advice from other academics and invited a consultant from CEPH to review the existing programme and comment on what was needed to pursue accreditation as a public health programme. This visit occurred as CEPH was in the process of revising its criteria; the discussion focused on planning to meet the likely new criteria [35]. Several content and procedural shortcomings were identified, including lacking well-formed internship and capstone activities, requiring too few credits and having several gaps in the required curriculum. The lack of formally trained public health faculty in sufficient numbers and disciplinary diversity to merit accreditation also was noted.

### Prioritize within constraints and imperatives

With this information in hand and a positive assessment of support within the college and university administration, the Department began planning to implement the community roundtable's three recommendations. Implementing these recommendations involved a set of planning activities for faculty and administrators alike. Those deliberations, in turn, stimulated iterative efforts between the college, the university and external stakeholders to generate additional information, clarify and develop a shared vision and identify issues for further deliberation and future consideration.

In 2003, planning commenced to implement the first recommendation: to transform the MS in Health Promotion to a CEPH-accreditable MSPH programme. The Department identified the faculty and other resources needed to implement the new programme and a coordinator for the new programme was recruited, as were other faculty. Consensus for the direction and focus of the new MSPH emerged. Acting on the second recommendation – to plan for a school of public health – would require more time and would need to await the arrival of additional public health-trained faculty. The third community recommendation – the establishment of an advisory board – was immediately implemented. The newly created Public Health Advisory Board reflected an intentional mix of leaders from local health departments, large health care organizations and wellness-oriented community agencies.

### Set primary goals/objectives

Based on these development priorities, two mandates emerged: hire more faculty, emphasizing those with training in core public health disciplines; and begin the complex academic planning and curriculum development process. In 2005, the department of five full-time faculty members doubled with the addition of three junior faculty members as well as two senior faculty members with institution-wide responsibilities. Two more faculty members joined these ranks in 2006, four in 2007 and three in 2008, with two more expected for the 2009/2010 academic year.

With this growing cadre of faculty, work on the planning and implementation of these degree programmes moved forward with increasing speed. Academically, the first order of business was to reshape the existing MS in Health Promotion into an MSPH programme. A detailed curriculum proposal and justification, building on the material collected as part of the SWOT analysis and the community roundtable, was presented to the university governance for approval. Concurrently, students in the soon-to-be-supplanted MS programme were advised of the planned changes and involved in the transition process.

### Specify educational strategies/approaches

The goals of the new MSPH required an integration of professional practice and research competence within a 45-credit, 21–28-month programme. These objectives dictated an intensive academic programme complemented by practical learning in both field and research/development settings. Educational strategies appropriate for the specific disciplines and courses were recommended, with careful attention to cross-cutting and capstone activities.

### Establish processes for defining, assessing and revising curriculum

While the university and college had established processes for reviewing and approving curricula, the Department, due to its heretofore small size, had always acted as a "committee of the whole" with minimal oversight of the faculty responsible for coordinating specific programmes. The department's expanding faculty complement and portfolio of programmes necessitated the formation of a departmental curriculum committee (consisting of the coordinators of the various programmes) supported by ad hoc faculty advisory committees for each programme. The ad hoc committees were replaced in Fall 2007 with the newly formed Public Health Programs Governance Committee and expanded in Fall 2008 to include programme-specific support committees. This model provided the requisite support and oversight to speed and improve the quality of the tremendous amount of academic planning needed to implement and administer these programmes.

### Bottom-up

With the specific tasks for the faculty defined in terms of key objectives and features, the bottom-up portion of the process commenced.

### Define competences

As the curriculum proposal to revise the MS in Health Promotion into an MSPH moved through the university governance system, efforts were initiated to more explicitly define the competences and assessment methods needed to ensure that the totality of the curriculum provided what was needed to produce competently trained graduates. Sources such as the Association of Schools of Public Health (ASPH) competence project as well as competences developed by other schools and programmes were consulted [36,37]. These ideas, as well as those coming from the faculty and the advisory board, were adapted to the specific mission, vision and circumstances of the UNC Charlotte community. Consensus emerged about the optimal capabilities and preparation of a UNC Charlotte MSPH graduate, the most appropriate conceptual model for organizing and depicting the competences and the type of student the programme should target [38].

### Select an organizing framework/paradigm

An examination of the UNC Charlotte MSPH competence listing in combination with reviewing the approaches of other MSPH and MPH programmes led to an organizational approach that mirrors the MSPH programme's dual emphasis on research and practice. The 45-credit curriculum (requiring two to two-and-a-half years of full-time study) was organized such that students concurrently follow a professional practice (problem-solving) sequence integrated with a research process sequence: students are exposed to epidemiology and biostatistics concurrently with research methods; and to behavioural and environmental determinants of health concurrently with programme planning and programme evaluation.

**Figure 4:**
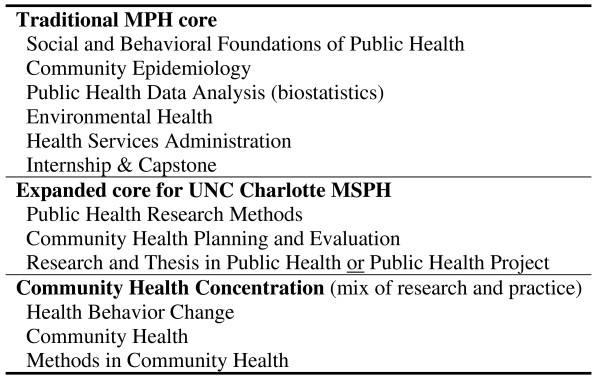
**UNC Charlotte MSPH curriculum**.

The sequence first delivers the core curriculum (expanded beyond that typical of MPH programmes) followed by courses designed to facilitate the integration and application of the new knowledge and skills in both practice and research contexts (Figure 4). The practicum develops competence in professional practice. The capstone thesis or project develops student competence in applying research skills through mentored research (thesis) or evidence-based public health practice (project) experiences that result in a scholarly paper appropriate for an academic or professional audience. These core training experiences are coupled with coursework that develops greater depth of knowledge within a given area. Coordination efforts ensure that classes appropriately address both practice and research implications of contemporary public health topics.

### Define the curriculum

Meshing the delivery of these competences into the organizational approach for the MSPH programme led to a validation (with minor tweaking) of the revised core and focal area curricula [39]. Course content was organized and sequenced to provide to students the requisite competence to be successful graduates, where success is defined by the community's stated needs and expectations and by evolving professional standards (e.g. employer type and employment rates, pass rates on credentialling examinations, competitiveness for doctoral programmes and contributions to professional practice).

### Relate curriculum to competences

A concurrent process to organizing the competences into a coherent curriculum of courses and learning experiences was the development of a matrix to ensure that students were indeed provided training on and were assessed for mastery of all the explicit competences. The curriculum-competence matrix ensured there were no areas where competence was expected without a defined (formal or informal) training opportunity. Likewise, the matrix helped identify areas where competences might be better sequenced or where explicit course prerequisites were needed to ensure students had acquired basic competence before more advanced skills were taught. This review also helped ensure that any duplication of competence was planned reinforcement and review rather than unintended redundancy.

Once completed with the core of the MSPH, the process was extended to its focal areas (somewhat akin to a concentration or track), then to the planned BSPH major. This planning process is now integrated as a routine part of the Department's curricular planning activities.

### Align competences and student assessment Methods

Once assured that the curriculum was providing the requisite competences and that these competences were being formally assessed, attention shifted to the assessment methods themselves. Assessment methods naturally reflect the traditions of the various disciplines that comprise public health. Methodological disciplines such as epidemiology and biostatistics typically use problem-based exams and case studies, while behavioural sciences often use essay-based and oral examinations and professional programmes rely on internships, capstone experiences and other practical demonstrations. Far too often, administrative ease or a lack of understanding results in inappropriate assessment tools being used for a given situation [40] (i.e. it is exceedingly difficult to assess a student's ability to critically synthesize and apply knowledge in a "real world" situation by means of a multiple-choice exam).

Ensuring congruence between a competence and its assessment tool, therefore, requires concerted effort on the part of the faculty to examine assessment at both a course and a programmatic level: it may be fine to assess only a student's ability to calculate a chi-square statistic on a biostatistics end-of-class exam if the student will have to demonstrate the ability to determine which statistics are appropriate for a given set of data elsewhere in the curriculum. Integration, application and practice objectives are one area where practica and capstones (theses or projects) can serve a critical catch-all role, in much the same way as qualifying exams for doctoral students. External assessments, such as student performance on credentialing exams such as the CHES (Certified Health Education Specialist) or the newly launched CPH (Certified in Public Health), can also provide timely feedback on the adequacy of a programme's preparation of its graduates [7,8].

### Reassess goals

As the bottom-up portion of the process reaches its conclusion, the information gathered during the prior steps then feeds back into the assessment of the mission, to begin the next iteration of the cycle. The first task was to ensure that what emerged from the cycle adequately responded to the needs generated by the top-down process. Course, exit, employer and alumni surveys and other assessments provided useful and timely feedback that informed programmatic change. When deviations in the articulation between the processes were identified, the faculty determined how best to reconcile these differences and assess whether the deviation were due to flaws in the application of the process or reasoned responses to an ever-changing environment. Identification of new threats and opportunities is a useful by-product of this process, and one that often stimulates new and innovative lines of inquiry that will in turn stimulate new directions in the strategic assessment.

The first iteration of the process at UNC Charlotte led to changes in the sequencing of core courses (to better support students as they now begin to engage in their thesis project in parallel to their coursework) and to end the cross-listing of two core courses (to provide a smaller class size and a greater focus on the specific needs and interests of public health students). At a faculty level, this process also led to a commitment to work toward dual CAHME (Commission on Accreditation of Healthcare Management Education) and CEPH accreditation of the Department's MHA programme as one part of the larger initiative to evolve toward status as a school of public health.

This expanded vision of public health offerings at UNC Charlotte has been shared with the University Administration and is now being shaped through a participatory stakeholder process, restarting the cycle of strategic planning and engagement that brought the Department to this point. In autumn 2008, growth of the UNC Charlotte Public Health Programs into a school of public health emerged as one of the campus's priorities during the UNC system-wide "UNC Tomorrow" strategic planning process [41].

## Discussion and Evaluation

It is only through this rigorous and cyclical process of determining what society needs, designing a curriculum specifically to prepare graduates to meet those needs, ensuring that those graduates meet those needs and reassessing society's needs that we can continue to advance the profession and ensure the public's health. In the case of the Department of Public Health Sciences at UNC Charlotte, the most recent cycle has been transformational and exceptionally fast.

**Figure 5:**
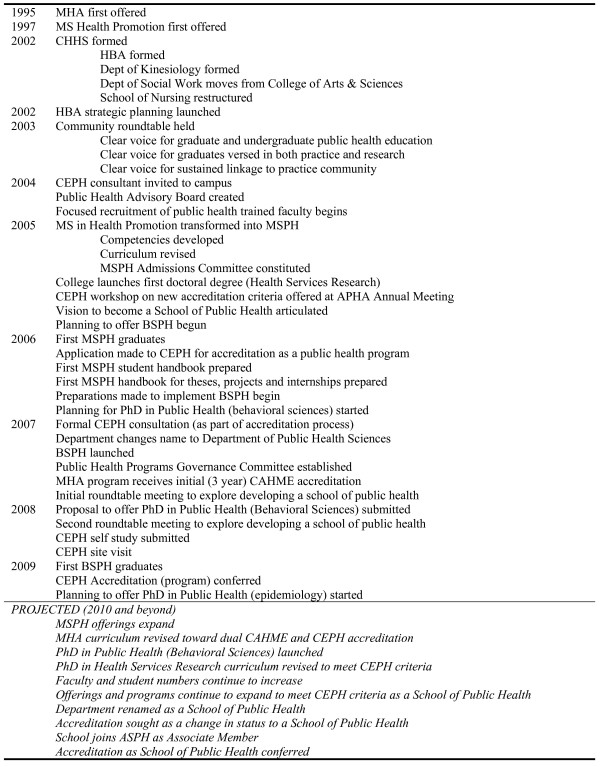
**Achievements and projected timeline**.

As summarized in Figure 5, the Department has had a string of rapid successes and ambitious plans for attaining status as a school of public health in the next decade. Notable achievements include the graduation of the first MSPH class in the spring of 2006, designation of attaining school of public health status as a campus priority in Fall 2008, graduation of the first BSPH class in the spring of 2009 and awarding of initial CEPH accreditation as a public health programme in June 2009.

This rapid progress was made possible by the strategic allocation of human and capital resources from across the university and by the stakeholder support for student internships and other facets of the programme that relied on community engagement. This campus and community support was itself a product of these strategic planning efforts that had defined, justified and effectively conveyed the value of these initiatives to key decision-makers at critical times in the planning process.

## Conclusion

By following and trusting the process, UNC Charlotte has made great strides in a short time. The programme is by no means assured of success and cannot claim that it is a model worthy of emulating other than by these short-term successes. Still, several lessons have emerged thus far. These lessons are not new or innovative, but they bear repeating:

• Be connected to your community.

• Know your community and its needs.

• Be responsive to your community.

• Build coalitions by building consensus.

• Remember that in the long run, process is as important as product.

• Plan inclusively and effectively.

• Recognize and seize opportunities.

• Prepare for success.

The adaptable, empirically derived, top-down/bottom-up strategic planning process described here provides a structured, comprehensive and inclusive process for ensuring that these lessons are grounded into organizational practice.

It is a constant challenge to keep advancing on all the fronts needed to meet this new vision for the future while ensuring that the infrastructure and community support are in place. It is a delicate balance between moving too quickly and making costly errors versus moving too slowly and losing critical momentum and public support or missing out on opportunities entirely. This dilemma is captured by the new buzzword in urban development: 'smart growth'. The challenge we face as academic and professional programmes in public health is growing as quickly as possible to meet the vast unmet community need, but in a way that ensures the sustainability, effectiveness and efficiency of the programme. Following a strategic planning, community engagement and curricular design process such as the one outlined above is one means to ensure that curricula are responding to a community's needs and ultimately improving the public's health.

## Competing interests

The authors declare that they have no competing interests.

## Authors' contributions

MET conceived of the paper and its organizing principles; AH & MET implemented the strategic planning process described in the paper; ME, a practitioner stakeholder, guided the conceptualization and implementation of the process and assisted with the analysis and interpretation of the results. All authors read and approved the final manuscript.
